# TypOn: the microbial typing ontology

**DOI:** 10.1186/2041-1480-5-43

**Published:** 2014-10-18

**Authors:** Cátia Vaz, Alexandre P Francisco, Mickael Silva, Keith A Jolley, James E Bray, Hannes Pouseele, Joerg Rothganger, Mário Ramirez, João A Carriço

**Affiliations:** INESC-ID, R. Alves Redol 9, 1000-029 Lisboa, Portugal; Instituto Superior de Engenharia de Lisboa, Instituto Politécnico de Lisboa, R. Cons. Emídio Navarro 1, 1959-007 Lisboa, Portugal; Instituto Superior Técnico, Universidade de Lisboa, Av. Rovisco Pais 1, 1049-001 Lisboa, Portugal; Instituto de Microbiologia, Instituto de Medicina Molecular, Faculdade de Medicina, Universidade de Lisboa, Av. Prof. Egas Moniz, 1649-028 Lisboa, Portugal; Department of Zoology, University of Oxford, Oxford, UK; Applied Maths NV, Keistraat 120, 98308 Sint-Martens-Latem, Belgium; Ridom GmbH, Mendelstr. 11, D-48149 Münster, Germany

**Keywords:** Ontology, Knowledge representation, Microbial typing methods

## Abstract

**Abstract:**

Bacterial identification and characterization at subspecies level is commonly known as Microbial Typing. Currently, these methodologies are fundamental tools in Clinical Microbiology and bacterial population genetics studies to track outbreaks and to study the dissemination and evolution of virulence or pathogenicity factors and antimicrobial resistance. Due to advances in DNA sequencing technology, these methods have evolved to become focused on sequence-based methodologies. The need to have a common understanding of the concepts described and the ability to share results within the community at a global level are increasingly important requisites for the continued development of portable and accurate sequence-based typing methods, especially with the recent introduction of Next Generation Sequencing (NGS) technologies. In this paper, we present an ontology designed for the sequence-based microbial typing field, capable of describing any of the sequence-based typing methodologies currently in use and being developed, including novel NGS based methods. This is a fundamental step to accurately describe, analyze, curate, and manage information for microbial typing based on sequence based typing methods.

## Introduction

It is widely known that different strains from a given bacterial species may have distinct phenotypic behaviors, such as a higher capacity to cause invasive disease, to asymptomatically colonize the host or to present resistance to antimicrobials [1]. Such distinguishing characteristics can be usually attributed to lineages identified at the level of the genotype. Microbial typing refers to the methodologies used to identify these lineages and define them at sub-species level. Microbial typing has important implications in several health related fields such as surveillance of infectious diseases, outbreak investigation and control, identification of pathogen reservoirs, and studies on pathogenesis [2]. Traditionally these methodologies were based on characterizing a limited number of markers. These markers can be phenotypic characteristics, such as the presence of certain structures on the bacterial surface [3], or genetic characteristics, such as the presence on the bacterial genome of DNA sequences that are recognized and cleaved by specific enzymes, generating band patterns by gel electrophoresis [4]. More recently, due to the low cost and increasing availability of DNA sequencing technologies, the development of typing methods became focused on the use of DNA sequence information.

Although these methods revolutionized microbial typing, through the creation of novel, unambiguous and easily understandable nomenclatures for human use, the existing databases still lack interfaces for machine-readable formats, which can be used for automated data submission and querying. An ontology describing the concepts and relationships for sequence-based typing methods can thus provide a powerful tool in the field. Sharing data annotated in a common language, and in a semantically rich machine-readable format, will allow a better integration of the data existing in databases of sequence-based typing methods, epidemiological information and the novel NGS data being produced [5]. Furthermore, it can facilitate comparison of different typing schemas, and allow users to mine, in an effective way, the ever-growing public data.

In this paper, we describe the design of TypOn, a microbial typing ontology. TypOn was developed from a previous prototype ontology [6], and focuses on sequence-based typing methods, including novel NGS methodologies. We discuss the connection of TypOn to existing ontologies, how to use it to annotate data already publicly available, and the methods to effectively query it.

### Domain description

Several typing methods have been used in outbreak detection and epidemiological surveillance ranging from phenotypic methods to fragment based methods and sequence based methods [5, 7].

Multilocus Sequence Typing (MLST) [8, 9] is a widely adopted methodology to type several diferent species of microorganisms. This method is based on determining the sequence of internal fragments of multiple (typically seven) loci encoding proteins with housekeeping functions. By a locus we understand a specific location in the chromosome, where different sequences occupying a given locus define distinct alleles of that locus. Each set of loci defining an MLST scheme is proposed by a group of researchers that usually also provide evidence supporting its discriminatory power and suitability for its intended purpose. When applying the methodology, the set of alleles identified at the loci considered define a sequence type (ST), a key identifier with this methodology. The loci chosen are usually different for each species, although some species may share some or even all loci in their MLST schemas. The number of loci can be also variable and can be greater or smaller than the seven loci more commonly adopted. MLST’s large appeal for the community was the reproducibility and portability of results, which allowed the deployment of databases for several bacterial species [10–12]. The strain nomenclature developed by MLST facilitated the global tracking and immediate comparison of microbial strains in clinical and research settings.

Another sequence-based method that derived its success from a common nomenclature and the ease of strain classification, was *spa* typing for *Staphylococcus aureus*[13], an important pathogen that is one of the major causes of nosocomial infections [14]. This method is based on repeat sequences present at a single locus, the *spa* gene. These repeats are short sequences of DNA (in the case of *spa* about thirty nucleotides) that, although sharing consensus characteristics, can be variable in their sequence. Their number is also variable and, in the case of *spa* typing, this is expressed by a string of numbers representing the identity and number of repeats present at each *spa* allele. An updated list of identified repeats and *spa* types can be found at http://spaserver.ridom.de/.

Multilocus Variable Number of Tandem Repeats Analysis (MLVA) [15] is a method that is based on the number of repeat patterns present on several defined locus that are, similarly to MLST, defined in a schema. Several schemas are also available in multiple websites such as http://www.mlva.eu/, http://www.mlva.net/ or http://www.miru-vntrplus.org/.

With the advent of Next Generation Sequencing (NGS) technologies, and the ability to produce a draft genome sequence of a microbial strain in a couple of days instead of weeks or months, researchers can use this information to classify the strain according to new or previously available sequence-based typing methods. Furthermore, novel typing methods are being developed that are able to probe tens, hundreds or even thousands of different loci across the genome [16], effectively extending the MLST concepts. An example of such a method is ribosomal MLST [17]. Other whole genome methodologies probe the genome for Single Nucleotide Polymorphisms (SNPs) when compared to reference genomes [18, 19] in order to discriminate strains.

The main goal of typing methods is the characterization of individuals existing in a given sample. The sample under study can be recovered from sick or healthy subjects or directly from the environment. The first process is the isolation of the microorganisms to be characterized from the sample collected. Each individual, or in the case of bacteria, each colony isolated from the microbial population then becomes an “isolate”, referring to the process of isolating it from a sample that contains many microorganisms potentially representing distinct species. In particular each isolate can then be identified at subspecies, species, or genus level, identifying it as belonging to a given taxonomic unit, i.e., taxon. Moreover, since most microorganisms reproduce asexually the subsequent propagation of this isolate in the laboratory as an axenic culture, would be expected to generate a clonal population.

Each isolate can be associated with typing information and ancillary details. The isolate can have the nucleotide sequence of its genetic material determined and multiple typing characteristics defined through different typing methods, such as MLST, *spa* typing or MLVA [15]. There are several categories of typing methods. For instance, although MLST and *spa* typing are both genotypic methods, MLST is a multi-locus typing method while *spa* typing is a single-locus typing method.

For the specific case of multi-locus typing methods, several schemas have been defined as indicated above. Each schema is characterized by the (possibly ordered) set of loci selected for a given taxon, usually defined at the species level. Each schema is then administrated by one or a group of microbiologist, or by an institution, and each isolate’s ancillary data and respective typing information are deposited in public repositories and validated by a curator. Different schemas can be defined for the same taxon and an ontology will facilitate understanding the relationships between different schemas. Ancillary data include information about the place where the microorganism was isolated, information about environment or host, and other possible contextual details. Later on, we will discuss how this information can be added and annotated in the context of TypOn.

We also note that data ownership is a particular delicate issue in the surveillance of communicable diseases and, as we will discuss later, the approach proposed in this paper allows a straightforward implementation of the agreed upon policies. The sensitive nature of the information and the ethical issues associated can be also safeguarded through the application of suitable access control levels based on the ownership structure embedded in the ontology.

## Microbial typing ontology

TypOn is an OWL ontology for describing microbial typing, focusing on sequence based methods. Such a description will provide a universal framework for the exchange of information on the microbial typing field, allowing the integration of data coming from the numerous and disparate online databases.

In the next Section, we describe and discuss the TypOn ontology and its suitability for describing knowledge in the microbiology typing methods domain. Later, we illustrate how to annotate and query microbial typing data and information using TypOn.

### Ontology modeling

**Figure 1:**
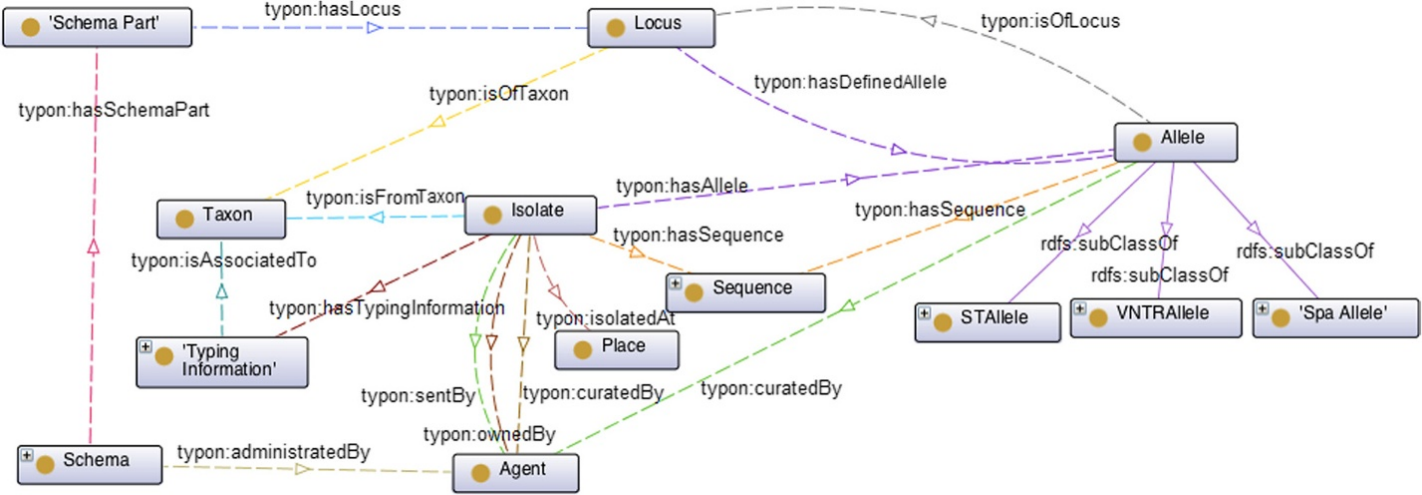
**The** ***Isolate*** **concept and its object properties.** Dashed lines represent object properties and solid lines represent subclass relations, e.g., *STAllele* *is-a* *Allele*. Properties *hasLocus* and *isOfLocus* have cardinality of exactly one. All other properties do not have any cardinality restriction.

**Figure 2:**
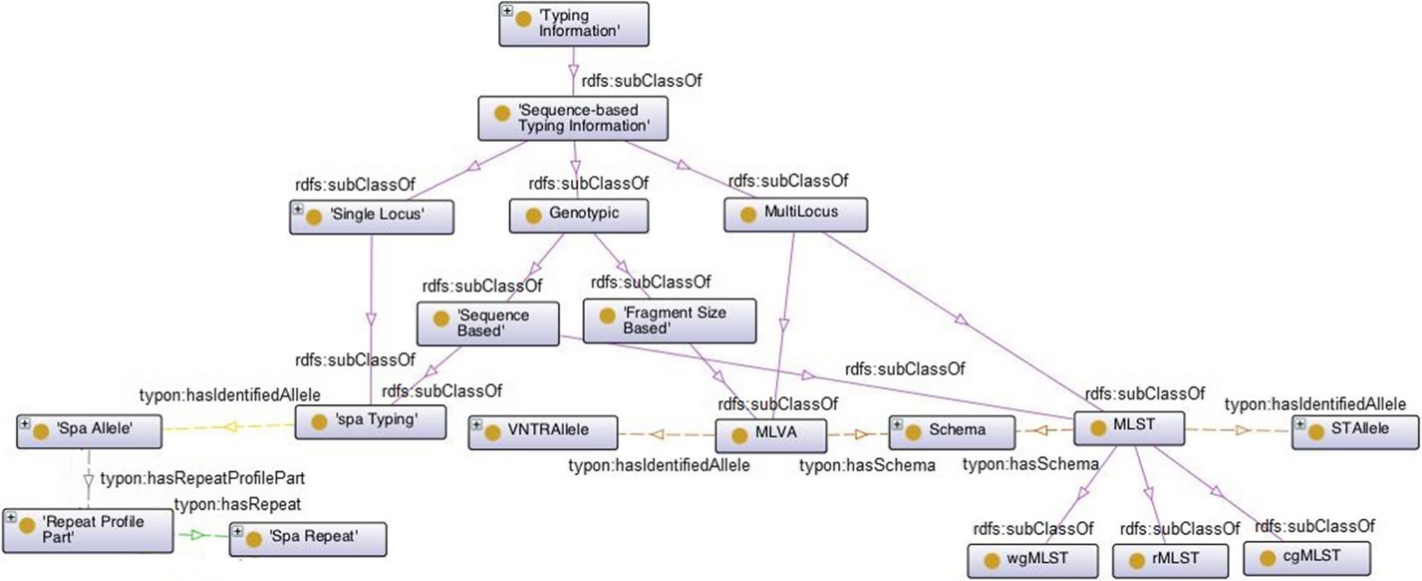
**Typing Information hierarchy and its object properties.** Dashed lines represent object properties and solid lines represent subclass relations, *e.g.*, *Genotypic* *is-a* *Sequence-based Typing Information*. Property *hasSchema* has cardinality of exactly one. All other properties do not have any cardinality restriction.

The main concepts and properties defined in TypOn are depicted in Figures 1 and 2. The ontology, which is an extended version of a previous prototype ontology [6], is available at http://purl.phyloviz.net/ontology/typon. In this current version, we added new concepts and refined existing ones, based on comments made by domain experts, including microbiologists and industry partners. The aim of TypOn is to represent knowledge about any of the currently used sequence-based microbial typing methods. TypOn can be reused as well as expanded, whenever new requirements and new technologies demand it. For backwards compatibility, older versions can still be used by explicitly stating the TypOn version. For instance, version 20140606 may be accessed and referenced as http://purl.phyloviz.net/ontology/20140606/typon. This ontology has been developed in the context of the Patho-NGen-Trace project http://patho-ngen-trace.eu/project/ as a way to standardize microbial data exchange between online databases and current software using those databases.

Ongoing developments, new versions, as well as use cases and examples, can be found in the project repository https://bitbucket.org/phyloviz/typon, and documented in the project wiki https://bitbucket.org/phyloviz/typon/wiki. The base URL for TypOn, http://purl.phyloviz.net/ontology/typon, redirects transparently to the last stable version of the ontology, in RDF/XML format, located in the master branch in the development repository.

The current version of TypOn has 44 classes, including those imported from other ontologies, and 47 properties, as shown in Table 1.

**Table 1:** **Statistics concerning concepts and properties either defined in TypOn or reused from other ontologies/vocabularies**

Ontology	Concepts	Object	Data
		properties	properties
Microbial Typing	24	21	25
Ontology (TypOn)			
Basic Formal	8	1	0
Ontology (BFO) [20]			
Sequence Ontology	6	0	0
(SO) [21]			
Environment Ontology	2	0	0
(EnvO) [22]			
Ontology for Biomedical	1	0	0
Investigations (OBI) [23]			
Uniprot Core	1	0	0
Ontology (UNIPROT) [24]			
Friend of a	1	0	0
Friend (FOAF) [25]			
The DBpedia	1	0	0
Ontology [26]			

Although we will discuss examples of bacterial typing, we believe that TypOn is equally applicable to typing methods used to characterize any microorganism. Naturally, this implies that other loci, in addition to those used in the examples, will have to be entered into the ontology and new schemas will have to be defined. The ontology offers a flexible framework with which the existing and future sequence typing methods can be described and the examples are meant to illustrate its application as well as its flexibility. As described above, the first process in microbial typing is the recovery of the microorganisms to be characterized from the sample collected and, thus, *Isolate* is a main concept in TypOn and it is characterized by several properties.

Figure 1 shows an overview of the *Isolate* class and its related concepts and properties.

Each *Isolate* when identified at subspecies, species, or genus level, belongs to a certain *Taxon*, a relation that we express through the property *isFromTaxon*. The *Taxon* concept is reused from the Uniprot Core Ontology [27] for classifying life forms. Moreover, we define that each *Isolate* is an *Organism*, a concept that is reused from the Ontology for Biomedical Investigations (OBI) [28]. We also know the *Place* from where each *Isolate* was recovered, which we describe through the property *isolatedAt*. As with the *Taxon* concept, the *Place* concept is reused from another ontology, in this case the DBpedia ontology [29]. One can also define the environment material or system where a given Isolate was collected. These concepts are already found in the environment ontology (EnvO) [22] and, hence, we reused them and we add the properties *isolatedOnMaterial* and *isolatedOnSystem*, relating these concepts with the concept Isolate. Each *Isolate*, can have *Sequence* information, *i.e.* the nucleotide sequence of its genetic material, and *Typing Information*. The property *hasTypingInformation* commonly has cardinality higher than one for each *Isolate*, since several typing methods, such as MLST or MLVA, can be applied to the same *Isolate*. Later we will present an example of an isolate with both MLST and *spa* typing information.

In this context, it is important to note that *TypingInformation* is the root of a class hierarchy (see Figure 2). This hierarchy can be extended by including new, and already known, typing techniques, such as phenotypic information related to antibiotic susceptibility. In particular, we are able to distinguish different categories of typing methods. Let us consider *MLST* and *spa Typing*. Both are *Genotypic* methods, but the first is a *Multilocus* method while the latter is a *Single Locus* method.

**Figure 3:**
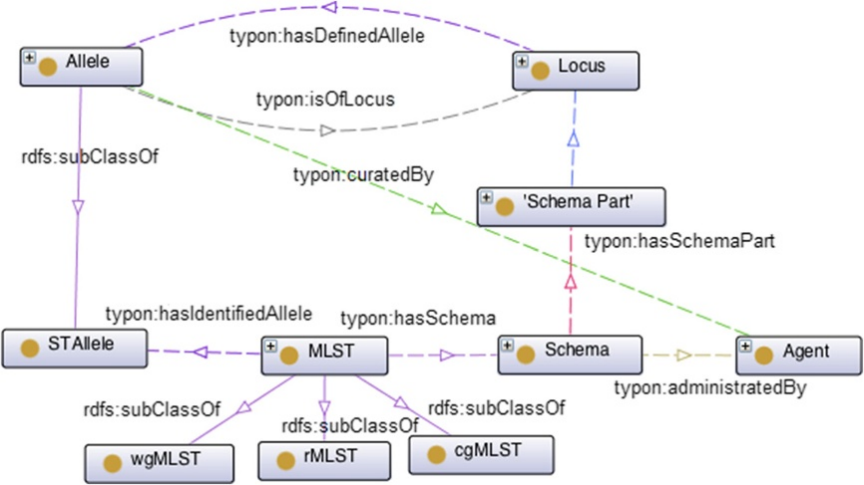
**MLST typing information.** Dashed lines represent object properties and solid lines represent subclass relations. Property *isOfLocus* has cardinality of exactly one. All other properties do not have any cardinality restriction.

Let us consider the concepts *Locus*, *Allele*, *Schema* and *MLST*, as depicted in Figure 3. In *MLST* we can have several typing schemas administrated by some *Agent*, a concept reused from FOAF ontology [25], for instance the database curator, and composed by a set of *Schema Parts*. Such schemas are represented through the class *Schema*, which has associated the property *hasSchemaPart*. The *Schema Part* concept allows us to identify a particular *Locus* and provide the index order for that locus in the underlying schema through object property *hasLocus* and data property *index*, respectively. In the case of *MLST*, each locus identifies a region within the coding sequence of an housekeeping gene. Thus, the object property *hasLocus* associated to the *Schema Part* concept has cardinality of exactly one.

As depicted in Figure 1 and indicated above, each *Isolate* may have been characterized by more than one typing method. In the case of MLST, this kind of typing information can be subject to different schemas, resulting in different sequence types, which are characterized by the alleles found at each locus. Therefore, in our ontology, we associate to each *MLST* both a schema and a set of observed alleles through properties *hasSchema* and *hasIdentifiedAllele*, respectively. The property *hasSchema* has cardinality of exactly one and, hence, *MLST* and *MLVA* instances must be related with one and only one given *Schema*. Notice that *STAllele* is an *Allele* (see Figure 1) defined in a MLST Schema, disjoint from *Spa Allele* and *VNTAllele*. So, we only associate to *MLST* typing information the concept of *STAllele*. This is necessary given the different ways in which the distinct alleles are defined in these typing methods.

The entity *Spa Allele* is used in the context of *spa typing*. Each *spa typing* information has a matching *spa Allele*, which corresponds to a specific sequence of repeats found as a result of the amplification of the locus of the *spa* gene of *Staphylococcus aureus*. In Figure 2 we can observe that a given *Spa Allele* has *repeat profile parts*, *i.e.*, an entity that stores the index order of a given *Spa Repeat* in a given *Spa Allele*. Notice that each *Spa Repeat* may occur more than once in a given *Spa Allele* and that different *Spa Alleles* can have distinct number of repeats.

Notice also the difference between properties *hasIdentifiedAllele* and *hasDefinedAllele* relating respectively *MLST* and *Locus* concepts with the *Allele* concept. One could imagine that alleles associated to a given *MLST* instance could be obtained through the defined *Schema*, since property *hasDefinedAllele* allows to relate loci and alleles. But this is not the case. A *Locus* may have associated many alleles, with each of them belonging to many *MLST* instances, and hence we cannot identify the allele belonging to a particular *MLST* instance. That can be accomplished through property *hasIdentifiedAllele* which relates each *MLST* instance with its identified alleles. Although we did not add those kind of assertions in our ontology, we can still use this information to assert that the identified alleles for a given *MLST* instance are sufficient against a given *Schema*. We could even infer for which schemas a given *MLST* instance provides enough information.

It is also important to note that, by knowing only the *Locus*, it is possible to identify the *Taxon* that it belongs to, using the *isOfTaxon* property (see Figure 1).

Additional information for each class, such as *sample collection date* and *other id*, are described through data properties. For instance, the class *Allele* has data properties such as *id* and *date entered*. The class *Isolate* has data properties such as *sample collection date* and *date entered*.

Whenever possible, we reuse concepts from and establish relations with other ontologies as indicated in Table 1. An *Agent* is a concept imported from the FOAF ontology [25] and it can be a person, a group of persons or even an institution. In TypOn it allows the description of a person or a group of persons who have curated the information about the isolate, who have submitted that information to the database and who own the isolate. These relations are described by the object properties *curated by*, *sent by* and *owned by*, respectively. Reusing the *Agent* concept is extremely useful because it will allow, for instance, the use of TypOn together with the *Web Access Control* ontology [30] for defining access control levels in microbial typing databases, an important issue as mentioned above. Several applications in defining access control have been widely discussed and are well known to the research community [31, 32].

All TypOn concepts were derived from the Basic Formal Ontology (BFO) [20], the Ontology for Biomedical Investigations (OBI) [23] and the Sequence Ontology (SO) [21] to ensure upper-level interoperability with other ontologies. In order to avoid huge imports in TypOn we have used OntoFox [33] to query and import only relevant concepts in top level ontologies. These are then imported as, and are available at, https://bitbucket.org/phyloviz/typon/raw/master/imported.owl.

TypOn was also submitted to the BioPortal (https://bioportal.bioontology.org/), hosted by the National Center of Biomedical Ontologies (NCBO), being also available at http://bioportal.bioontology.org/ontologies/TYPON.

**Figure 4:**
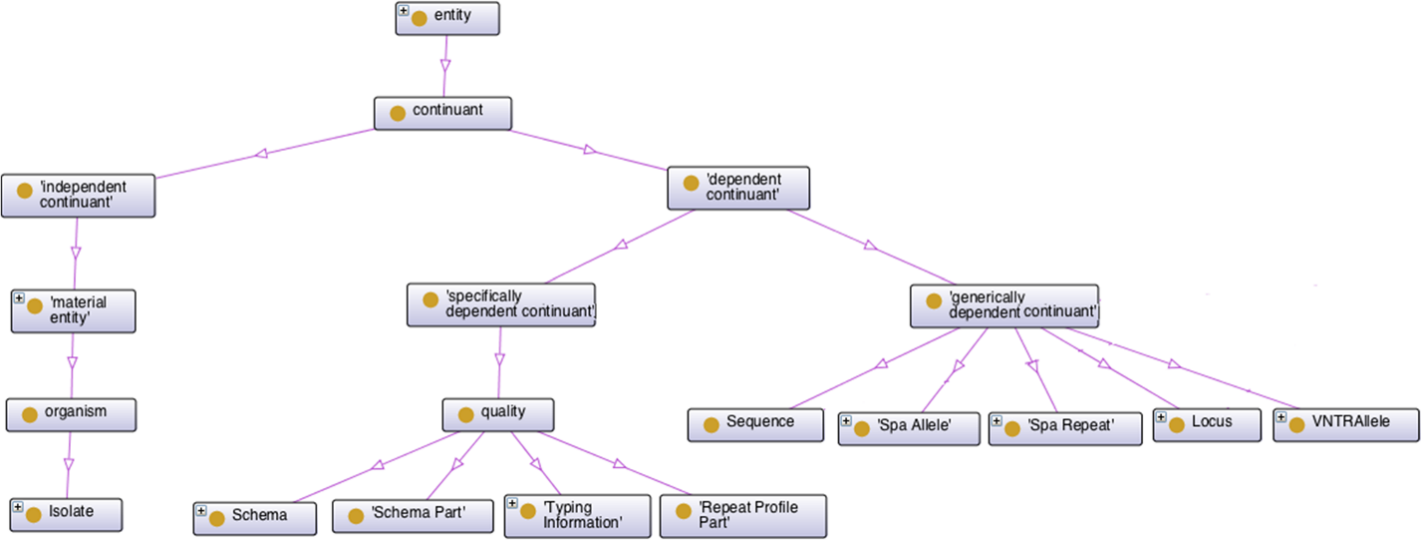
**Relationships among TypOn, the Basic Formal Ontology (BFO), and the Ontology for Biomedical Investigations (OBI).** The lines represent subclass relations.

Concepts such as *Schema* and *Typing Information* are qualities (BFO _0000019), i.e., a categorical property. As discussed, we have classified an *Isolate* as an *organism* (OBI _0100026). More details in Figure 4.

**Figure 5:**
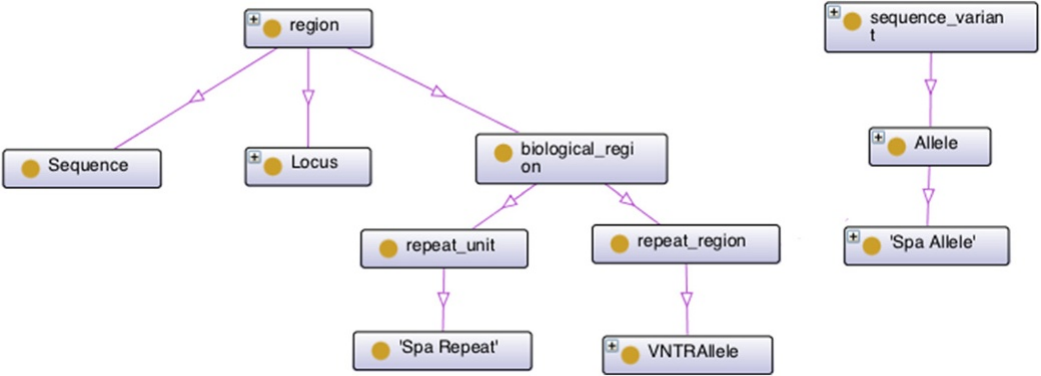
**Relationship between TypOn and the Sequence Ontology (SO).** The lines represent subclass relations.

Figure 5 depicts the Typon concepts that are related to the Sequence Ontology. Notice that we define *Locus* as a *region* (SO:0000001), since it is a named region on a genome.

Another example is the *Spa Repeat* concept which is a *repeat unit* (SO:0000657). Both *Locus* and *Spa Repeat* are also *generically dependent continuant* (BFO _0000031) since *region* (SO:0000001) and *repeat unit* (SO:0000657) concepts are subclasses of BFO _0000031 as defined in the sequence ontology (SO). Thus these concepts are related to both SO and BFO (see Figures 4 and 5).

### Use case

In this section, we illustrate how we can represent typing information annotated with the TypOn ontology. Our example makes use of data regarding the characterization of a *Staphylococcus aureus* isolate for the purpose of this case. We will use the Turtle language [34] in the description of our isolate.

**Figure 6:**
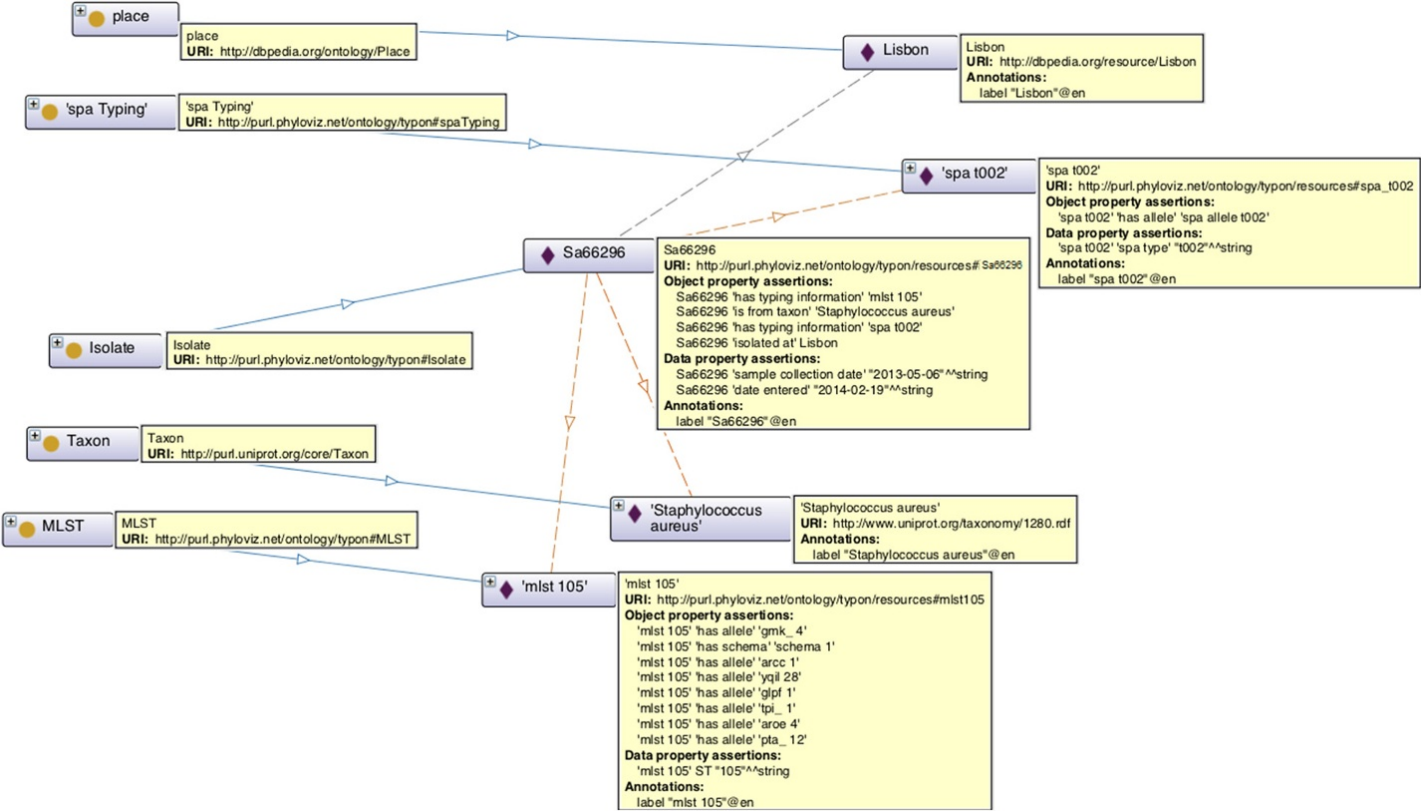
**The isolate** ***Sa66296*** **and its object and data properties.** Dashed lines represent object properties and solid lines represent subclass relations. Described properties do not have any cardinality restriction.

We can represent the isolate named *Sa66296* as follows:


This is an instance of *typon:Isolate* labelled as *“Sa66296”*. *rdfs:label* is an instance of *rdf:Property* that may be used to provide a human-readable version for the name of a resource. We further specify that it has two pieces of typing information *mlst105* and *spa _t002* (instances of *typon:MLST* and *typon:spaTyping*, respectively). Thus, these pieces of typing information are other individuals annotated with our ontology. We can keep track of the dates when the isolate was collected and when the isolate was entered into the system using properties *sampleCollectionDate* and *dateEntered*, respectively. We also describe the origin of the isolate, with the individual labelled *Lisbon* which has type *dbpedia-owl:Place*. Note that this isolate was recovered in Lisbon, Portugal, represented as a resource in DBpedia. Figure 6 depicts the isolate information.

**Figure 7:**
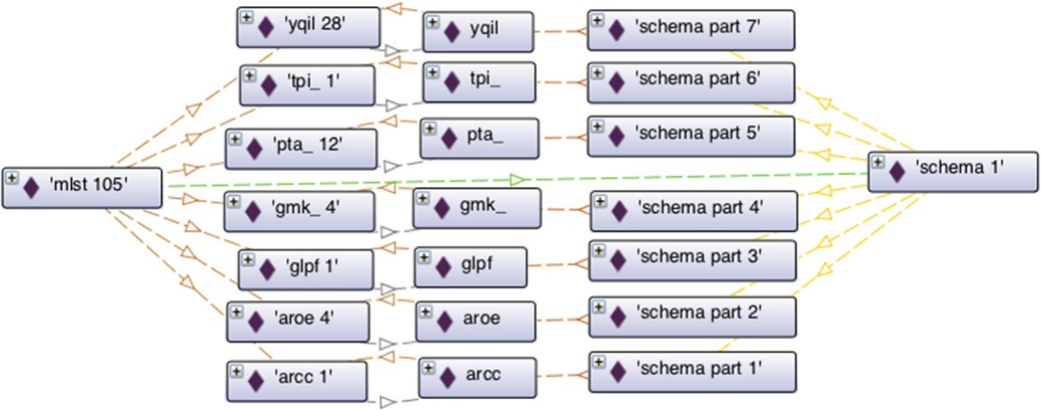
**MLST typing information for isolate** ***Sa66296*** **.** Dashed lines represent object properties. Instance *mlst 105* of *typon: MLST* is related with instance *schema 1* of *typon: Schema* through object property *typon: hasSchema* with cardinality of exactly one. Instance *mlst 105* is also related with several instances of *typon: Allele*, e.g., instance *aroe 4*, through *typon: hasIdentifiedAllele*. Instance *schema 1* is related with several instances of *typon: SchemaPart*, e.g., instance *schema part 2*, through property *typon: hasSchemaPart*. Each instance of *typon: SchemaPart* is related with an instance of *typon: Locus*, e.g., instance *schema part 2* is related with instance *aroe*, through property *typon: hasLocus* with cardinality of exactly one. Each instance of *typon: Allele* is related with an instance of *typon: Locus*, e.g., instance *aroe 4* is related with instance *aroe*, through property *typon: isOfLocus* with cardinality of exactly one. Each instance of *typon: Locus* is also related with an instance of *typon: Allele*, e.g., instance *aroe* is related with instance *aroe 4* through property *typon: hasDefinedAllele*.

The individual labelled *mlst 105* represents the sequence based typing method with a schema defined by the sequence of seven housekeeping loci, represented as follows:


Note that the individual labelled *schema1* identifies the seven housekeeping loci, using individuals of type *typon:SchemaPart* for keeping track of the index of each locus:


Let us now describe the individual that represent the *arcc* locus, the first locus in the considered schema as described by the individual labelled *schema_part_1*:


Note that the identified alleles of the loci can be directly obtained from the individual labelled *mlst105*. Furthermore all possible defined alleles can be obtained from the respective loci. It is also possible to obtain the locus that is associated to each allele, namely by property *typon:isOfLocus*. Figure 7 summarizes the representation of the MLST typing information concerning the isolate in our example. The complete example is available at https://bitbucket.org/phyloviz/typon/raw/master/test/Sa66296.ttl.

## Annotating data

In this section we discuss how to annotate a large dataset with the TypOn ontology and how to perform queries. We started by writing a D2RQ mapping for the data available for *Neisseria spp*, one of the databases available in our local BIGSdb [16] installation. D2RQ [35, 36] is a mapping language and platform for treating non-RDF relational databases as virtual RDF graphs, aiming to expose RDBs on the semantic web. The mapping allows us to reuse existing vocabularies and ontologies, such as TypOn, to map relational schemas, such as the one underlying BIGSdb. We note that our mapping is not exhaustive and that we just annotated part of the data with TypOn ontology. The mapping is available at https://bitbucket.org/phyloviz/typon/raw/master/test/BIGSdb_d2r_mapping.ttl.

Even though the D2RQ web app provides a SPARQL endpoint, it turns out that queries may take a long time to complete, causing high loads in the underlying database, and the web app may also become irresponsive. To overcome this issue we used the tool dump-rdf available with D2RQ to dump all triples and we uploaded them to a local instance of Virtuoso, which among other functionalities includes a highly efficient triple store (http://virtuoso.openlinksw.com/). Hence, a more responsive SPARQL endpoint is available at http://data.phyloviz.net/sparql, where we should select http://rest.phyloviz.net/neisseria/ as the default graph. All resources at http://rest.phyloviz.net/neisseria/ are also dereferenceable through rewrite rules against the SPARQL endpoint.

Let us consider a few SPARQL queries for illustration purposes. Imagine that we wanted to define a new MLST schema that includes loci *carB*, *glnA*, and *rpiA*. How can we find all isolates for which we already have typing information under this new schema? It turns out that we can answer this question with a SPARQL query:


We can then submit this query to our endpoint at http://data.phyloviz.net/sparql and our results include:


As another example, we may be interested in exploring the variability at the third locus in any MLST schema in our dataset, but only for isolates of *Neisseria polysaccharea* found in Canada. Taking into account the relationships defined in TypOn and Uniprot, we can retrieve this variability as follows through a federated query:


By submitting this query to http://data.phyloviz.net/sparql, we obtain


where the prefix *db:* stands for http://rest.phyloviz.net/neisseria/resource/.

## Final remarks

TypOn provides the basic concepts needed to establish the vocabulary and the semantic relationships for different sequence-based typing methods, and it is designed to allow further expansion. It was defined based on three different approaches to sequence-based typing: using the DNA sequence information directly, using the sequence of repeats in a DNA sequence, and for MLVA, using the number of repeats in a locus. Since these three approaches can be used to define many of the existing typing methods, TypOn can be easily expanded to encompass the newer multilocus typing techniques that are appearing based on NGS technologies, defined by expansion of the MLST concepts to larger numbers of genes [37] or by Single Nucleotide Polymorphism approaches, where each position on the genome can be viewed as a locus and the nucleotide present as an allele. Other advantages of this ontology is that it can provide a consistent link with legacy microbial typing techniques and provide a way to describe and annotate the evolution of specific typing schemas. This will be of paramount importance, if schemas that will be constructed by grouping loci from existing schemas or adding new loci, are to be designed and represented in an accurate way. This ontology is the first stepping stone on the implementation of a semantic web approach for the data repositories in this field. It lays the foundation for a common language that can be used to integrate and link data from different typing databases and for a complete merging of microbial typing with microbial genomics. Using the strategy discussed in the previous section (Annotating data), a SPARQL endpoint is already deployed for the Pubmlst MLST databases at http://pubmlst.org/sparql. This endpoint accesses data annotated using TypOn for MLST databases for 75 distinct bacterial species that are hosted at Pubmlst.org and further 29 species hosted externally to Pubmlst.org. A RESTful API is also being developed to facilitate data access without requiring the SPARQL endpoint. Future work will focus on expanding the ontology and creating and deploying RESTful APIs to perform not only custom querying but also automated submission and curation of data for authenticated users, in order to speed up and distribute the curating process, and ensure better quality and reproducibility of data in the field of microbial typing.
